# Association Between Twitter Mention and Open-Access Status on Article Citation Metrics in the Field of Ophthalmology

**DOI:** 10.7759/cureus.31048

**Published:** 2022-11-03

**Authors:** David F Santos, Nasir Asif, Gabriel F Santos Malave, Natalio Izquierdo

**Affiliations:** 1 Ophthalmology, School of Medicine, Medical Sciences Campus, University of Puerto Rico, San Juan, PRI; 2 Medicine, Rutgers University, Newark, USA; 3 Medicine, Icahn School of Medicine at Mount Sinai, New York City, USA; 4 Surgery, School of Medicine, Medical Sciences Campus, University of Puerto Rico, San Juan, PRI

**Keywords:** ophthalmology, citation metrics, open access, social media, twitter

## Abstract

Introduction: It is possible that social media use can boost not just articles’ social impact but the number of citations and academic influence as well. If a positive correlation between Twitter usage and citation metrics exists in the ophthalmology literature, it is important to broadcast this information to the ophthalmology community so they can use Twitter to increase academic engagement with their research. There has also been an increase in the number of articles available as open access. Therefore, it is important to evaluate the presence of an open-access citation advantage in the field of ophthalmology. This study aims to evaluate the relationship between Twitter mention and open access status on citation metrics in the ophthalmology literature.

Methods: We conducted a retrospective cross-sectional study comparing article citation metrics to Twitter mentions and open access status. We gathered data on ophthalmology research articles from the six highest-ranked ophthalmology journals published as part of a January 2019 issue. Data were collected in April 2022, 38 months after online publication. Data on citations for each article was based on Google Scholar and Scopus websites. The Altmetric Bookmarklet extension was used to determine the amount of social engagement each article received. The open-access status of each article was based on the status listed in its corresponding journal. Two-tailed t-tests were used to compare social media engagement and open access status with the number of Google Scholar and Scopus citations.

Results: A total of 102 original research articles were analyzed. 89 (87.3%) articles received a Twitter mention. Articles tweeted at least once had a significantly higher Google Scholar score (27.2 +/- 4) compared to articles not tweeted (16.4 +/- 1.7; 1.7-fold increase, p<0.05). Likewise, the average Scopus score was significantly higher for tweeted articles (18.6 +/- 2.6) compared to articles not tweeted (11.8 +/- 1.6; 1.6-fold increase, p<0.05). Articles listed as open access had a significantly higher number of Twitter mentions (11.8 +/- 1.8) compared to articles that were not open access (5.6 +/- 0.7; 2.1-fold increase, p<0.05). Open-access articles also had higher citation scores compared to articles that are not open access, but this relationship was not statistically significant.

Conclusion: This is the first study to evaluate the relationship between article Twitter mention and citation score in the field of ophthalmology. It demonstrates a significant positive correlation between the article Twitter mention and citation score and provides further evidence that social media engagement can be beneficial to the dissemination of academic information. Further studies on the relationship between social media engagement and article dissemination are warranted in the field of ophthalmology.

## Introduction

The academic impact of scientific research articles has traditionally been gauged through the number of citations for a said article in peer-reviewed journals [1]. Given the rise and increased usage of social media, social media impact score has emerged as an alternate metric to assess academic impact [2]. Altmetrics is a system that provides a score based on online mentions of scientific publications and can be used to assess the social impact of an article [3].

It is possible that social media use can boost not just articles’ social impact but the number of citations and academic influence as well. There has been documented evidence that an author’s Twitter usage can be used as a predictor for citation metrics for publications [2]. Further, specialty-specific research has shown that the number of Twitter mentions positively correlates with publications’ citation numbers [4-8].

Since each specialty has embraced social media usage at different rates, the relationship between social media and citation numbers should be evaluated for each specialty [9,10]. If a similar relationship in ophthalmology literature between Twitter usage and citation metrics exists, it is important to broadcast this information to allow members of the ophthalmology community to know that they can use Twitter to increase academic engagement of their research results. 

Moreover, there has been an increase in the number of articles available as open access in recent years [11]. Open-access articles are freely available to the public and therefore their open-access status could facilitate academic engagement. Research on the existence of an open-access citation advantage has yielded mixed results [12]. Therefore, it is important to evaluate the presence of this advantage in ophthalmology.

Previous studies in the field of ophthalmology have evaluated the relationship between social media and journal impact [13,14]. However, to the best of our knowledge, there has been no research exploring the relationship between social media and citation numbers for articles in the ophthalmic literature. Likewise, no research has explored the existence of an open-access citation advantage in the field of ophthalmology.

Given that social media usage (including Twitter) has been increasing among ophthalmologists (and academic ophthalmology), we believe there exists a positive relationship between Twitter mentions and ophthalmology citation metrics [15-17]. We believe this positive relationship will be similar to what has been reported in other surgical subspecialties [4,5]. Due to the increased accessibility of open-access articles, we also believe there exists a positive relationship between open-access status and ophthalmology citation metrics.

## Materials and methods

We conducted a retrospective cross-sectional study comparing article citation metrics to Twitter mentions and open access status. The analysis of the association between Twitter mentions and citation metrics has previously been outlined in studies in urology and otolaryngology [4,5]. Since this study did not involve patients, Institutional Review Board approval was not required.

Article selection

Data on ophthalmology research articles from six different journals published as part of a January 2019 issue were reviewed. This date was selected to provide enough time for dissemination and citation of the articles across media to take place. A previous study in the field of otolaryngology also used the January 2019 issue of journals for article selection [5]. Another study in the field of urology also limited the data collection period to one month; therefore, to maintain consistency, we chose to only analyze journal publications from a one-month period, January 2019 [4]. Likewise, in the previously reported methodology, journals were selected based on Scimago journal and country rank; the top journals were selected because they were expected to have the most social media attention [4,5]. Due to our goal of comparing our findings with previous studies in other surgical subspecialties we sought to maintain as much consistency as possible with the previously reported methodology [4,5]. Therefore, the top six journals based on Scimago journal and country rank 2021 which had a January 2019 issue with original research articles were selected. We decided to include six journals because we believed they would provide a large enough sample size for analysis. The following journals were included: Ophthalmology, Ocular Surface, American Journal of Ophthalmology, Retina, JAMA Ophthalmology, and British Journal of Ophthalmology. The inclusion criteria for articles were original research articles, published as part of a January 2019 issue. Exclusion criteria included: research letters, brief reports, editorials, and any other publication not considered original research articles. The decision to exclude all publications which are not original research articles was also made to maintain consistency with the previously reported methodology [4,5].

Data selection

Data were collected in April 2022, 38 months after online publication. Data on citations for each article were based on Google Scholar and Scopus websites. Article titles were searched on each website to determine the number of citations each article had received. The Altmetric Bookmarklet extension was used to determine the amount of social engagement each article received. We gathered data on Twitter mentions for each article. Further, we recorded how long it took for an article to be mentioned on Twitter after it was either published online or published in print. Categories for a timeline from online publication included < 1 week online, < 1 month online, and 1 month to print date.

Categories for a timeline from print publication included < 1 week in print, < 1 month in print, and < 1 year in print. Twitter mentions which occurred after one year were not reported to maintain consistency with similar studies that have demonstrated no increase in Twitter mentions after one year of print publication [4,5].

Cross-references of Twitter handles with authors to determine if a Twitter mention was from an author of the paper mentioned were done. Articles tweeted by one of its authors were listed as an author's self-tweet. The open-access status of each article was based on the status listed in its corresponding journal.

Data analysis

Data were analyzed using SPSS software (IBM Corp., Armonk, NY). Significance was set at p-value <0.05%. Two-tailed t-tests were used to compare the presence or absence of Twitter mentions with the number of Google Scholar and Scopus citations. Levene’s Test for Equality of Variances was used to test for homogeneity of variance and the corresponding t-test result was used. The same analysis was implemented with regards to the presence or absence of author self-tweet. A two-tailed t-test was used to compare the number of citations and Twitter mentions between articles that were listed as open access and those which were not open access.

## Results

A total of 102 original research articles were reviewed. Table 1 shows the citations, tweeted articles, Twitter mentions, Google Scholar score, and Scopus score for each journal included.

The median Google Scholar score (IQR) and the median Scopus score (IQR) were 16.5 (15.5) and 10.5 (12), respectively. Eighty-nine (87.3%) articles received a Twitter mention. The median number of Twitter mentions was 5 (6). The journal with the highest median Google scholar and Scopus scores was Ophthalmology with scores of 29 (40.5) and 23 (26.5), respectively. The journal with the highest median number of Twitter mentions was JAMA Ophthalmology with 15 (4).

**Table 1:** Average metrics by journal ^a^Values are presented as number; ^b^Values are presented as median (IQR); ^c^Values are presented as number (%)

Journals	Articles^a^	Google Scholar Score^b^	Scopus Score^b^	Tweeted articles^c^	Twitter mentions^b^
Ophthalmology	14	29 (40.5)	23 (26.5)	14 (100)	10 (4.3)
Ocular Surface	15	17 (10)	11 (9)	8 (53.3)	5 (5.25)
American Journal of Ophthalmology	19	14 (12.5)	10 (8.5)	19 (100)	5 (3)
Retina	25	14 (17)	10 (13)	19 (76)	2 (1)
JAMA Ophthalmology	9	20 (24)	13 (20)	9 (100)	15 (4)
British Journal of Ophthalmology	20	13 (12.3)	7.5 (8.5)	20 (100)	5 (3.3)
Total	102	16.5 (15.5)	10.5 (12)	89 (87.3)	5 (6)

**Figure 1:**
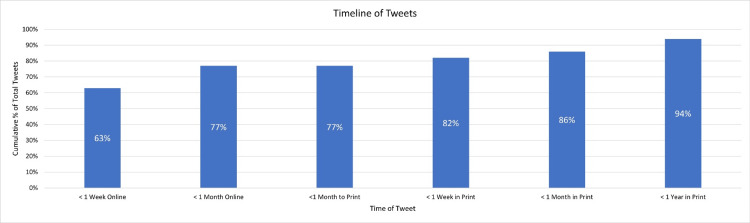
Twitter mention timeline

Figure 1 depicts the timeline of Twitter mentions. Two articles did not have the timeline of their Twitter mention listed and were excluded from the analysis. The highest number of tweets occurred less than one week after online publication (62%) and 77% of tweets occurred before print publication. Almost all Twitter activity occurred within one year of publication (94%).

Table 2 demonstrates the two-tailed t-test results comparing citation scores between articles that received at least one Twitter mention and those which were not tweeted. It also includes a comparison between articles self-tweeted by authors versus no self-tweet. Articles tweeted at least once had a significantly higher Google Scholar score (mean ± SE of 27.2 ± 4) compared to articles not tweeted (16.4 ± 1.7; 1.7-fold increase, p<0.05). Likewise, the average Scopus score was significantly higher for tweeted articles (18.6 ± 2.6) compared to articles not tweeted (11.8 ± 1.6; 1.6-fold increase, p<0.05). No significant relationship was identified with regard to the author's self-tweet.

**Table 2:** Average citation scores relative to the presence of Twitter mention and author self-tweet *All citation scores presented as mean ± SE

Metric	Not tweeted (n=13)	Tweeted (n=89)	P-value	No Author tweet (n=98)	Author tweet (n=4)	P-value	No News mention (n=94)	News mention (n=8)	P-value
Google Scholar	16.4 ± 1.7	27.2 ± 4	<0.05	26.3 ± 3.7	13.3 ± 4.3	>0.05	20.6 ± 2	87 ± 33.3	>0.05
Scopus	11.8 ± 1.6	18.6 ± 2.6	<0.05	18.1 ± 2.3	9 ± 3.6	>0.05	14.3 ± 1.4	58.1 ± 19.2	>0.05

Table 3 demonstrates the results of two-tailed t-tests comparing an average number of Twitter mentions and citation scores based on open access status. Articles listed as open access had a significantly higher number of Twitter mentions (mean ± SE of 11.8 ± 1.8) compared to articles that were not open access (5.6 ± 0.7; 2.1-fold increase, p<0.05). Open-access articles also had higher citation scores compared to articles that are not open access, but this relationship was not statistically significant.

**Table 3:** Average citation scores and Twitter mentions relative to open access status *Citation scores and number of Twitter mentions presented as mean ± SE

Metric	Open Access Status	Column1	P-value
	No (83)	Yes (19)	
Google Scholar	25.5 ± 4.3	27.1 ± 4.2	>0.05
Scopus	17.3 ± 2.7	19.9 ± 3.3	>0.05
	No (71)	Yes (18)	
Number of Tweets	5.6 ±0.7	11.8 ± 1.8	<0.001
	No (3)	Yes (5)	
News Mention	5.3 ± 3.4	5 ± 3.7	>0.05

## Discussion

We evaluated the relationship between a Twitter mention and citation score over a 38-month period. We identified a positive relationship between Twitter mentions and citation scores across both citation metrics (Google Scholar score and Scopus score). The increase in citation score among tweeted articles was about the same for both Google Scholar and Scopus scores (1.7 and 1.6-fold increase respectively). This finding is in accordance with a similar study in the field of otolaryngology which also found a 1.6-fold increase in Google Scholar score for articles with a Twitter mention [5]. A similar study in the field of urology [4] found an even greater increase in both Google Scholar and Scopus scores (2 and 2.3-fold increase respectively). This association does not imply causation. Articles that are more interesting to readers could have received more Twitter mentions and might have been cited more regardless of Twitter mentions. A previous study in the field of ophthalmology determined that the total number of tweets per journal was significantly positively correlated with journal rank and impact factor [14]. Therefore, our results provide further evidence that an increased social media presence can improve the visibility of ophthalmology academic articles.

The timeline of Twitter mentions was evaluated. Most Twitter mentions occurred within one week of online publication (62%). This is in accordance with the previously mentioned studies in the fields of urology and otolaryngology which also found most Twitter mentions occurred within one week of online publication [4,5]. This suggests the period immediately following online publication could be the most crucial for social media engagement. Interestingly, 77% of Twitter mentions occurred before hard copy print publication. This finding is also in accordance with previous research and suggests the ideal time for social media engagement might have concluded once the article is in print [4,5]. In our study, there was no increase in Twitter mentions less than one month before to print date. This differs from previous studies in Urology, and otolaryngology [4,5]. In our study, an increase in Twitter mentions occurs after hard copy print publication (77% to 82%). This finding suggests that print publications may further boost social media engagement once the online period has concluded.

The relationship between open access status and citation scores and Twitter mentions was also evaluated. Articles listed as open access had a significantly higher number of Twitter mentions than articles that were not open access. This suggests that articles that are open access and therefore more accessible may reach a wider audience resulting in a higher number of Twitter mentions. Previous studies on the relationship between open access status and citation metrics have yielded mixed results with respect to the existence of an open access advantage [12]. Multiple studies have demonstrated the open access advantage varies with respect to field and discipline [12,18]. In our study, citation scores were also higher for open-access articles, even though this had no statistical significance. This finding suggests an increased importance for the evaluation of an open-access advantage specifically in ophthalmology.

Finally, we sought to evaluate the relationship between the presence of author-self tweets and citation scores. No significant correlation between the presence of author self-tweet and citation score was found. This fact contrasts with previous studies which found a significant increase in citation scores for articles that had a Twitter mention by an author [4,5].

Limitations of our study include the inability to demonstrate a causal analysis as well as a small sample size for author self-tweet. Perhaps more data could be gathered on these metrics by including a larger number of articles in the analysis. Moreover, only top-ranked journals and original research articles were selected for analysis. This decision was made to maintain consistency with previously reported methodology and compare our findings to other surgical subspecialties while limiting the presence of other confounding variables [4,5]. However, the decision to only include top-ranked journals and original research articles limits the generalizability of our findings. Further studies on the relationship between social media engagement and article dissemination are warranted in the field of ophthalmology.

## Conclusions

This is the first study to evaluate the relationship between article Twitter mention and citation score in Ophthalmology. Our study demonstrates a significant positive correlation between article Twitter mention and citation score. It provides further evidence that social media engagement can be beneficial to the dissemination of academic information. It also suggests that the open-access status of an article may increase Twitter mentions and help increase an article’s social media engagement. Further studies on the relationship between social media engagement and article dissemination are warranted in the field of ophthalmology.
